# Changes in the mucosal barrier during acute and chronic *Trichuris muris* infection

**DOI:** 10.1111/j.1365-3024.2010.01258.x

**Published:** 2011-01

**Authors:** S Z Hasnain, D J Thornton, R K Grencis

**Affiliations:** 1 Wellcome Trust Centre for Cell-Matrix Research, University of ManchesterManchester, UK; 2 Manchester Immunology Group, Faculty of Life Sciences, University of ManchesterManchester, UK

**Keywords:** glycocalyx, glycosylation, goblet cell, mucin, *Trichuris muris*

## Abstract

The intestinal mucosal barrier, part of the innate immune defence, is responsive to the external environment and changes in response to infection. There is disparate evidence for the epithelial and goblet cell products within the intrinsic barrier being part of a response to resolve infection. We comprehensively analysed the changes of mucosal glycoconjugates during acute and chronic infection by utilising the *Trichuris muris* (T. muris) model. Transcription factors, atonal homolog 1 (Math-1) and SAM pointed domain containing ETS transcription factor (Spdef) were activated during acute infection, which promoted stem cell fate towards a secretory cell phenotype. The thickness of the intermediate barrier, the carbohydrate-rich glycocalyx, composed of cell surface mucins increased with exposure to T. muris, with an increase in Muc4, Muc13 and Muc17. Overall, hypersecretion of glycoproteins into the extrinsic barrier (mediated by IL-13) via the gamma amino-butyric acid-α3 receptor (GABA-α3), was observed during acute infection. Furthermore, altered glycosylation was observed during acute and chronic infection; mucins were more highly charged during acute infection than during chronic infection. This study readdresses the changes within the mucosal barrier, in particular in the cell surface and secreted mucins during acute and chronic nematode infection.

## Introduction

The intestinal mucosa forms a dynamic defensive barrier that is responsive to the external environment (1). The barrier has two major components: the intrinsic barrier that consists of the continuous epithelial cell lining and the extrinsic barrier that is the combination of the secretions from the goblet cells within the epithelium composing the mucus barrier (1,2). Moreover, cell surface mucins intercalated into the apical surface of the epithelium with their extracellular domain protruding out are a dominant glycoconjugate component of the glycocalyx, which forms an intermediate layer of defence between the extrinsic and intrinsic barriers (2). Different components of the mucosal epithelium have been extensively studied in relation to parasitic infections, particularly intestinal dwelling nematodes (3,4). It is clear that innate and adaptive immune systems play a key role in resistance and susceptibility to chronic nematode infections (5). Recent studies conducted on the nematode models have been focused on changes in non-mucin goblet cell products, such as resistin-like molecule-β and intelectin, in response to infection (6–8). Few studies, however, have fully characterised the prevalent glucoconjugate components of the goblet cells and the protective intestinal glycocalyx in response to nematode infection. In the light of the recent data demonstrating a clear role for Muc2, the major goblet cell product, during resistance to the mouse whipworm *Trichuris muris* (*T. muris*) (9), the present work has comprehensively characterised this goblet cell and glucoconjugate axis in relation to resistance or susceptibility to chronic infection.

The *T. muris* nematode survives in the host by eliciting a T Helper (T_H_)1-type immune response; however, resistance is dependent on a T_H_2-type immune response (10). In BALB/c mice, a high-dose infection (>150 eggs) results in the T_H_2-mediated expulsion of the nematode, whereas a low-dose infection (<15 eggs) results in a T_H_1-dominant immune response and consequently a chronic infection (11). Therefore, the nematode *T. muris* in mouse provides a manipulable system that can be used to investigate the changes in mucosal barrier operating during acute and chronic infection within a single system (10,12). It is thought that multiple effectors under immunological (T_H_2) control orchestrate the responses involved in the expulsion of the nematode (9,13,14). The intestinal epithelium, the niche of the parasite, consists of four distinct cells types; enterocytes, paneth cells, enteroendocrine cells and goblet cells (15). Stem cells at the bottom of the intestinal crypts can differentiate into absorptive cells (enterocytes) or secretory cells (paneth, enteroendocrine or goblet cells) (16). Absorptive cell differentiation is dependent on the transcription factor hairy and enhancer of split 1 (Hes-1), whereas differentiation towards secretory cell lineage is dependent on Math-1 (atonal homolog 1) (17). The recently discovered transcription factor, SAM pointed domain containing ETS transcription factor (Spdef), was shown to facilitate the terminal differentiation of secretory cells into goblet cells (16). During *T. muris* infection, within the niche of the parasite, hyperproliferation of cells in the intestinal crypts has been reported. Goblet cell hyperplasia has been described in models that are able to reject *T. muris* (9,18), whereas hyperplasia of enterocytes is observed during chronic infection (19). Goblet cell hyperplasia is thought to be largely under the control of T_H_2-type cytokines (20,21), although some studies have suggested that IL-4/IL-13 independent goblet cell hyperplasia can occur (22).

In this study, we aimed to conduct a comprehensive analysis of the changes in the components of the mucosal barrier: the epithelial cell layer, cell surface mucins and the extrinsic mucus barrier during acute and chronic infection. We reconfirmed that goblet cell hyperplasia is a prominent feature of acute infection as an activation of transcription factors involved in goblet cell differentiation was observed. Expression of cell surface mucins *Muc4*, *Muc13* and *Muc17* was elevated with the exposure to *T. muris* infection, resulting in an increase in the thickness of the apical glycocalyx. Interestingly, along with the increase in the storage and synthesis of glycoproteins within the goblet cells, which has been previously reported, an increase in secretion of these glycoproteins was observed during worm expulsion. The hypersecretion of glycoproteins was mediated by the gamma amino-butyric acid- α3 (GABA- α3) receptor under the control of the T_H_2-type cytokine, IL-13. Furthermore, we demonstrate for the first time that along with the depleted mucin production, there is a general alteration of glycosylation on the mucins in chronic infection.

## Materials and methods

### Animals

Male BALB/c mice were purchased from Harlan Olac. Female wild-type BALB/c mice, IL-4 Knockout (KO) (BALB/c-background) and IL-4R KO (BALB/c-background) mice obtained from Dr. McKenzie (20) were maintained in the Biological Services Unit at The University of Manchester. The protocols employed were in accordance with guidelines by the Home Office Scientific Procedures Act (1986). All mice (6–12 week old) were kept in sterilized, filter-topped cages, and fed autoclaved food in the animal facilities.

### Parasitological technique

The techniques used for *T. muris* maintenance and infection were described previously (23). Mice were orally infected with approximately 150 eggs for a high-dose infection and >15 eggs for a low-dose infection. Worm burdens were assessed by counting the number of worms present in the caecum.

### Histology, immunohistochemistry and immunofluorescence microscopy

The whole caecum (rolled) was fixed in 95% ethanol and processed by using standard histological techniques. Sections were treated with 0·1 m potassium hydroxide for 30 min prior to staining with Periodic-Acid Schiff’s (PAS). Slides were counterstained with 1% fast-green. Standard immunohistochemical and immunofluorescent staining methods (24) were used to determine the levels of Spdef using the spdef antibody (Abcam) and lectins (25). Biotinylated lectins (*Sambucus nigra*, *Mackia amuresis*, Peanut agglutinin, *Maclura pomifera*, *Dolichos biflorus* (DBA), *Triticum vulgaris* and *Ulex europeaus*) were used for the detection of glycans.

### Mucus extraction and agarose gel electrophoresis

The caecum was gently flushed with PBS and subsequently flushed five times with 3 m Urea to obtain the secreted mucus; the tissue was fixed for histology before and after treatment to ensure only the secreted mucus within the intestinal lumen was isolated without caecal crypt disruption (data not shown). The mucus was solubilised in 8 m guanidium chloride, reduced using 50 mm dithiothreitol and carboxymethylated using 0·125m iodoacetamide prior to electrophoresis on a 1% (w/v) agarose gel. Mucins were detected after western blotting with mucin-specific antisera (26). Subsequently, the staining intensity of the positive bands was determined using the GS-800 BioRad Densitometer (26,27).

### Anion-exchange chromatography

Crude mucus samples were reduced, carboxymethylated and treated with DNAse before being subjected to anion-exchange chromatography as described previously (28,29), using a Resource™ Q column (GE Healthcare, Buckinghamshire, UK). Samples were eluted with the starting buffer (10 mm piperazine at pH 5 in 6 m Urea containing 0·02% 3-[(chloamidopropyl) dimethylammonio]-1-propanosulphonate) for 15 min (0·5 mL/min), followed by a linear gradient (60 min) up to 0·4 m Lithium perchlorate-10 mm piperazine at pH 5 in 6 m Urea containing 0·02% 3-[(chloamidopropyl) dimethylammonio]-1-propanosulphonate (28,29). Fractions were blotted onto nitrocellulose membrane using a 72-well slot blot manifold (Schleicher & Schuell, Dassel, Germany) attached to a vacuum pump. Membranes were then assayed for glycoprotein using PAS reagent [as described previously (27)]. Staining intensity was measured using a Bio-Rad GS-800 Calibrated densitometer.

### Real time PCR (RT-PCR)

Total RNA from epithelial cells was isolated using the previously described method (14). cDNA was generated using an IMPROM-RT kit (Promega, Southampton, UK) and Absolute QPCR SYBR Green (ABgene, Epsom, UK) was used for quantitative PCR. Primer efficiencies were determined using cDNA dilutions and genes of interest were normalised against the housekeeping gene, β-actin and expressed as a fold difference to uninfected naïve mRNA levels. mRNA expression was investigated using the primer pairs shown in Table S1. Melting curves were analysed, and products were directly sequenced to verify the identity of the amplified genes. In brief, products were digested with Exonuclease I and Calf Intestinal Phosphatase and subsequently sequenced using the ABIPRISM Big-Dye Terminator cycle (Applied Biosystems, California, USA) sequencing reaction at the Sequencing Facility in The University of Manchester. The data were analysed using Chromas Pro v1.34, and the sequences obtained were compared against the GenBank database; http://www.ncbi.nlm.nih.gov/BLAST.

### Quantification of histological staining

The number of goblet cells was counted in 100 longitudinally sectioned crypt units (expressed per crypt). The number of epithelial cells was counted in 20 longitudinal crypts and expressed as per crypt. Five randomly selected fields of view were analysed to determine the levels of positive lectin staining, indicated as 90–100% (++++), 60–80% (+++), 10–50% (++) and 0–10% (+).

### Statistical analysis

All results are expressed as the mean ± SEM. Statistical analysis was performed using SPSS version 16.0. Statistical significance of different groups was assessed by using parametric tests; ONE-way Analysis of variance with post-test following statistical standards or paired student *t* test. *P <*0·05 was considered statistically significant.

## Results

### Up-regulation of secretory cell lineage transcription factors during acute *Trichuris muris* infection

It is well established that a T_H_1-type immune response (characterised by IFN-γ) leads to a chronic *T. muris* infection, whereas a T_H_2-type immune response (characterised by IL-4 and IL-13) leads to the expulsion of the nematodes (10). Chronic infection leads to changing the gross morphology of the niche of the parasite (caecum). Furthermore, no changes were observed in the number of goblet cell and, an increase in epithelial cell numbers was associated with chronicity (Figure S1).

**Figure 1:**
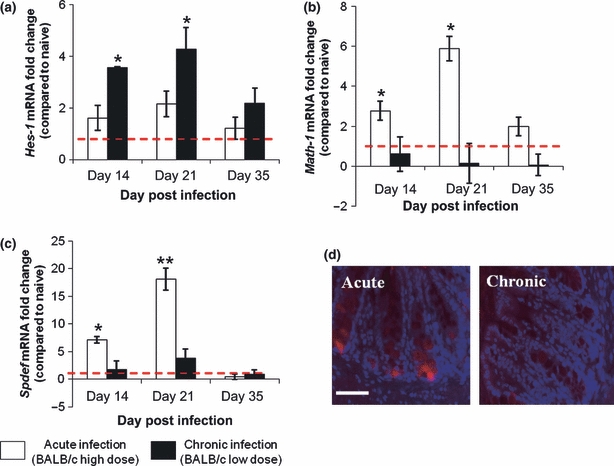
RT-PCR was used to determine the levels of *Hes-1* (a), *Math-1* (b) and *Spdef* (c) during acute (open bars) and chronic (closed bars) infection. Red dashed line = naïve levels. Immunofluorescence microscopy (d) was used to assess levels of Spdef on day 35 pi. Scale bar; 10 μm. *P < 0·05, **P< 0·01. Results represent the mean value of 5–10 mice per group ± SEM.

Stem cells located at the bottom of the intestinal crypts mature and differentiate into absorptive cells or secretory cells as migrate to the luminal surface (16). Therefore, to investigate whether there was a change in differentiation of stem cells that in turn promotes differentiation towards a certain cell lineage during acute or chronic infection, the expression of transcription factors involved in the differentiation of the intestinal progenitor stem cell into absorptive cells or secretory cells was determined using RT-PCR (Figure 1). The enterocyte differentiation transcription factor Hes-1, the transcription factor for differentiation towards absorptive cell lineage Math-1 (17) and Spdef, the transcription factor involved in the terminal differentiation of goblet cells (16) were chosen as markers of cell lineage. Levels of Hes-1 were significantly increased on day 14 and 21 post-infection (pi.), explaining the increase in epithelial cell numbers (Figure S1g), in chronic infection compared to acute infection and uninfected controls (Figure 1a). In contrast, Math-1 and Spdef were only significantly up-regulated on day 14 and 21 pi. of acute infection (Figure 1b, c) when an increase in goblet cell numbers was also observed (Figure S1h). Furthermore, immunofluorescence microscopy with the mSpdef antibody showed that Spdef was up-regulated at the bottom of the intestinal crypts of mice with acute infection (Figure 1d; on day 21pi.).

### An increase in glycocalyx thickness with exposure to *Trichuris muris* infection

**Figure 2:**
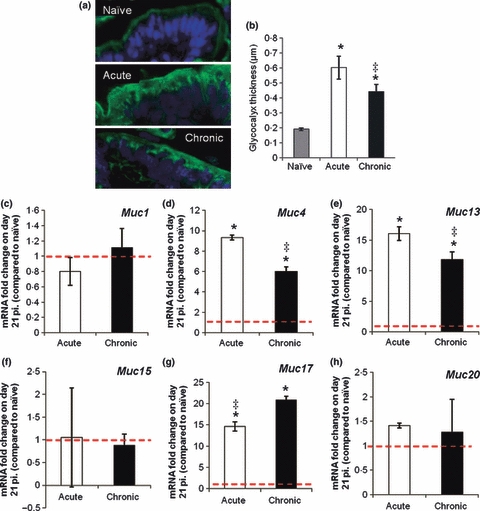
Changes in the glycocalyx were determined using immunofluorescence microscopy with the *Dolichos biflorus* lectin (a), the changes in the thickness were quantified (b). Levels of the cell surface mucins *Muc1* (c), *Muc4* (d), *Muc13* (e), *Muc15* (f), *Muc17* (g) and *Muc20* (h) were determined using RT-PCR during acute (open bars) and chronic (closed bars) infection. Red dashed line = naïve levels. * *P* < 0·05 compared to naïve. ‡ *P* < 0·05 compared to mice with acute or chronic infection. Results represent the mean value of 5–10 mice per group ± SEM.

The glycocalyx is the ‘carbohydrate-rich zone’ found at the surface of cells, consisting of glycoproteins including cell surface mucins that project from the apical surface of the cells (2). Immunofluorescence microscopy using the DBA lectin, which recognises the D-GalNAc glycan, revealed that with exposure to *T. muris* infection, there was an increase in the thickness of the glycocalyx layer, when compared to the uninfected naïve mice (Figure 2a, b). However, the glycocalyx was significantly thicker in mice during acute infection (day 21 pi.) when compared to during chronic infection.

To characterise the changes in the composition of the glycocalyx during acute and chronic *T. muris* infection, the levels of cell surface mucins in caecal tissue were determined using RT-PCR (Figure 2). In the intestine, *Muc1*, *Muc4*, *Muc13*, *Muc15*, *Muc17* and *Muc20* have previously been shown to be expressed (30). No major changes were observed in *Muc1*, *Muc15* and *Muc20* levels during chronic or acute infection when compared to uninfected control mice (day 21 pi. shown here; Figure 2). However, a marked increase in *Muc4*, *Muc13* and *Muc17* levels was observed in response to *T. muris* infection (Figure 2) and most likely explains the increased thickness of glycocalyx. The levels of *Muc4* and *Muc13* were significantly higher in the mice during acute infection; however, the increase in *Muc17* was more prominent in the mice during chronic infection. Similar trends in the levels of these cell surface mucins were observed on day 14 and 35 pi. (Table S2).

### Hypersecretion of glycoproteins mediated by GABA-α3 receptor during worm rejection

**Figure 3:**
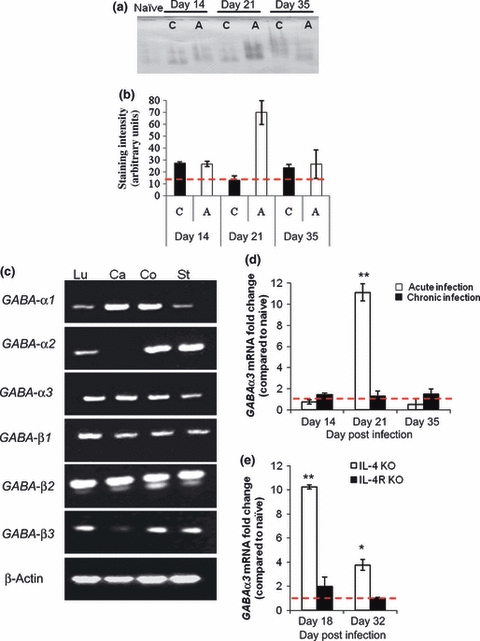
Mucus extracted into the intestinal lumen was isolated from mice with chronic (closed bars) and acute (open bars) infection on day 14, 21 and 35 pi. (pooled from five mice). Mucus was reduced and carboxymethylated, subjected to agarose gel electrophoresis, western blotted and stained with Periodic-Acid Schiff's (a) and staining intensity was measured (b). Representative three individual experiments. PCR was used to determine the basal expression of the gamma amino-butyric acid (GABA) receptor in a range of tissues; Lung (Lu), Caecum (Ca), Colon (Co) and Stomach (St). The levels of GABAα3 in the caecal tissue were determined post-infection during acute and chronic infection (d) or IL-4 KO and IL-4R KO mice (e). (Results represent the mean value of 3–8 mice per group ± SEM). Red dashed line = naïve levels. * *P* < 0·05. ** *P* < 0·01.

An elevation in transcription factors that determine the secretory cell lineage was observed during acute infection; furthermore, there was an increase in the number of goblet cells within the caecal crypts (Figure 1). Therefore, to determine whether there was also an increase in the secretion of glycoproteins during acute infection, the mucus secreted into the intestinal lumen was isolated and subjected to agarose gel electrophoresis (Figure 3a, b). On day 14 pi., an increase in the secretion of glycoproteins was observed in both acute and chronic models. Interestingly, during acute infection, as well as an increase in the amount of glycoproteins stored within goblet cells (refer to Figure S1), higher amounts of glycoproteins were secreted into the mucus barrier (Figure 3a, b).

Studies have shown that GABA-α receptors mediate the hypersecretion of glycoproteins into the mucus barrier, under the control of IL-13 in asthma models (31). Several studies have described GABA receptors, α-subunit 1–3 and β-subunit 1–3, to be expressed in the intestine (32,33). By utilising RT-PCR, we investigated the basal expression of these GABA receptors in the uninfected BALB/c mice; all GABA subunits, with the exception of GABA-α2 and GABA-β3, were expressed at high levels in the caecum (Figure 3c). Interestingly, in response to infection, only the expression of GABA-α3 was altered (Figure 3d); an increase in levels of GABA-α3 was observed during acute infection (day 21 pi.), and this correlated with the increase in glycoprotein secretion. The expression of all the other α and β isoforms remained unaltered when challenged with *T. muris* infection (data not shown). To dissect the role of the immune response in the up-regulation GABA-α3 receptor and mucus hypersecretion, we utilised the IL-4KO and IL-4R KO mice. The IL-4 KO mice do not contain any active IL-4 but are able to expel the nematodes because of IL-13-mediated effector mechanisms [Figure S2; (34)]. In contrast, the IL-4Rα1 chain is missing in the IL-4R KO mice and as both chains in the heterodimeric receptor are required to initiate intracellular signalling (35), IL-4 and IL-13 cannot act in the IL-4R KO mice, which are therefore susceptible to chronic infection [(34) Figure S2]. Interestingly, the levels of GABA-α3 remained unaltered in the IL-4R KO mice (Figure 3e), where goblet cell depletion was also observed (Figure S2). In the IL-4KO mice, however, along with the hyperplastic goblet cell response against *T. muris*, a 10-fold increase in levels of GABA-α3 was also observed in post-infection (Figure 3e).

### Altered glycosylation on goblet cell mucins during chronic *Trichuris muris* infection

**Figure 4:**
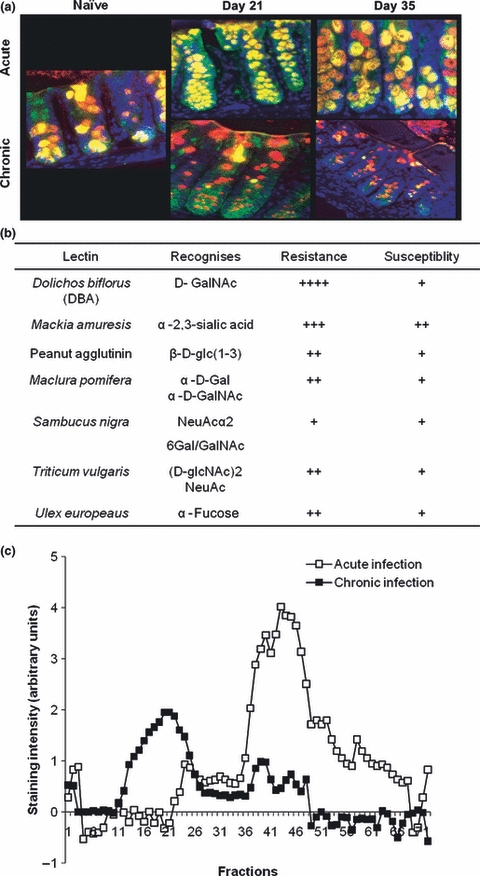
Dual immunofluorescent (a) staining with *Dolichos biflorus* (DBA) lectin (green) and mMuc2 antibody (red) in caecal tissue from mice with acute and chronic infection on days 21 and 35 pi. Merge (yellow) shows the co-localisation of Muc2 and lectins. (b) Quantitation scores of immunofluorescent staining of caecal tissue (Figure S3) with lectins (DBA, *Mackia amuresis*, Peanut agglutinin, *Maclura pomifera*, *Sambucus nigra*, *Triticum vulgaris*, *Ulex europeaus*) on day 21 pi. of acute and chronic infection. Secreted mucus was isolated from acute (open bars) and chronically (closed bars) infected mice on day 21 pi., reduced and carboxymethylated and subjected to anion-exchange chromatography. Fractions were collected, transferred to nitrocellulose membrane and stained with Periodic-Acid Schiff's (c). Results representative of three individual experiments.

Interestingly, a slight difference in the electrophoretic migration of mucins, when subjected to agarose gel electrophoretic analysis (Figure 3a), was noted during acute infection which suggested a change in glycosylation. Therefore, to evaluate the variation in mucin glycoconjugates in response to acute and chronic *T. muris* infection, dual immunofluorescence labelling for Muc2 (Muc2 antibody, red) and glycans (lectin, green) was performed (Figure 4a) and the relative lectin staining was quantified (Figure 4b). The most striking differences were observed in the staining with the DBA lectin that recognises the D-GalNAc glycan (Figure 4a). Most of the goblet cells within the caecal epithelium stained positive with the DBA lectin on day 21 pi. of acute infection, when the nematodes are rejected. In contrast, in the chronic infection, only a few goblet cells stained with the DBA lectin. During chronic infection (day 21 pi.), less staining was observed with all lectins employed (Figures 4a and S3). Low levels of staining were observed using the *S. nigra* lectin that detects the α-2,6-sialic acid compared to the *M. amuresis* lectin that detects the α -2,3-sialic acid. This suggested that mucins within the caecum contained mucins majorly containing the α -2,6-sialic acid.

Overall, lectin histochemistry analysis suggested that the mucins produced during acute infection are altered in their glycosylation compared to those produced by chronically infected mice. To assess whether the charge density of the mucins was also altered, the secreted mucus was isolated from the caecum of the two groups of mice on day 21 pi., reduced and carboxymethylated and analysed by anion-exchange chromatography (Figure 4c). PAS staining of fractions collected from the anion-exchange chromatography showed that mucins isolated from the chronically infected mice (on day 21 pi.) eluted before the mucins from mice with acute infection (Figure 4c). This suggested that the mucins in the caecum of the chronically infected mice were less charged when compared to the mucins from the mice with acute infection.

## Discussion

The *T. muris* nematode survives within its host by eliciting a T_H_1-type immune response in mice susceptible to chronic infection, whereas in an acute infection, a T_H_2-type immune response results in nematode expulsion (10). The intestinal mucosa, the niche of the nematode, forms a dynamic barrier that is responsive to the external environment and changes in response to infection because of the inflammatory response. It has already been demonstrated that T_H_2-type cytokine-mediated regulation of epithelial cell turnover (14), smooth muscle contractility (13) and the mucus barrier (9) can contribute to efficient worm expulsion. Recently, we have demonstrated a significant role for the intestinal mucin Muc2 (9), which is the major intestinal goblet cells product, during the expulsion of the *T. muris* nematode. In the present study, we aimed to characterise the changes within the mucosal barrier, in particular in its glycoconjugate components that may play a role in parasite rejection, by using the acute and chronic mouse models of *T. muris* infection.

Surprisingly, the glycocalyx that forms a layer of defence underneath the mucus barrier on apical surface of epithelial cells has not been investigated in this setting previously. Cell surface mucins, which are anchored to the apical cell surface, are a major component of this glycocalyx layer and extend further than most other cell surface structures (3,30,36–39). Cell surface mucins provide both a physical barrier and are also involved in intracellular signalling events; their expression has been shown to be both infection promoting and infection limiting with regard to pathogenic bacteria (2,3). We observed a striking difference in the thickness of the glycocalyx in response to *T. muris* infection, with the increase being more prominent during acute infection. Analysis of the cell surface mucin components, of the glycocalyx during acute and chronic infection, revealed an increase in the levels of *Muc4*, *Muc13* and *Muc17*, which most likely explains the increase in the thickness of the glycocalyx. Although the up-regulation of cell surface mucins was not exclusive to acute infection, they may contribute to worm expulsion. Whether this is because cell surface mucins directly influence the physicochemical properties of the glycocalyx or whether they are shed into the mucus affecting its barrier properties or have a role that is associated with the increased epithelial cell extrusion shown to be critical to worm expulsion (14), requires further attention.

The rejection of the whipworm was accompanied by increased differentiation of stem cells within the basal caecal crypts into goblet cells. Math-1 is involved in the differentiation of a progenitor stem cell into a secretory cell type, and Spdef subsequently regulates its terminal differentiation into a goblet cell (16,17). Both these transcription factors were up-regulated during acute *T. muris* infection, around the time of worm expulsion. In contrast, Hes-1, which is involved in the differentiation towards an enterocyte fate, was only up-regulated in the mice susceptible to chronic infection. This agrees with previously published data, where an increase in epithelial cell proliferation is observed during chronic infection (14,19). Overall, this clearly demonstrated that there is a bias towards goblet cell differentiation during acute infection which is not observed during chronic infection. The extrinsic mucosal barrier is composed of a mixture of products secreted by the goblet cells within the epithelial cell layer (40). Therefore, during acute infection, along with the increased differentiation towards the goblet cell lineage, and hence the increased levels of glycoproteins within the goblet cells, we also observed an increase in the amount of glycoproteins in the mucus barrier. The intestinal mucus barrier forms a bilayer: a ‘loose’ outer layer containing the commensal flora and the inner adherent layer that is devoid of commensal flora (41). It has been proposed that a diminished mucus barrier can result in inflammation in several mouse models (25,42,43), because of the direct contact of the commensal flora with the epithelial cell surface. We show here that there is a diminished glycoprotein content within the mucus barrier during chronic infection. Therefore, during chronicity to *T. muris,* the diminished mucus barrier could result in the commensal flora coming into contact with the epithelial cell layer, which may result in exacerbation of epithelial inflammation.

Previously published data have described goblet cell hyperplasia to be under the control of a T_H_2-type immune response. Although one study has shown goblet cell hyperplasia to be independent of IL-4/IL-13 (22), our data supported the hypothesis of T_H_2 cytokines controlling goblet cell hyperplasia post-*T. muris* infection (20,44,45). Studies have shown that GABA receptors expressed in the mucosal epithelium can mediate this hypersecretion of mucus in asthma (31). Our data demonstrated that within the caecum, GABA-α3 receptor expression is elevated in response to the increased IL-13 levels, which may mediate the secretion of glycoproteins into the mucus barrier. It is important to highlight that differences were observed in the expression of the GABA receptors between the caecum and the colon. This corroborates our previous findings where we observed goblet cell hyperplasia in response to *T. muris* only in the caecum (the niche of the parasite) (9). If in fact, the GABA-α3 receptor is up-regulated to mediate glycoprotein hypersecretion during worm expulsion, it could provide a novel therapeutic target that could be manipulated to induce the secretion of glycoproteins during chronic nematode infections, which would increase the thickness of the mucus barrier and potentially lessen inflammation.

Interestingly, lectin histochemistry has identified an alteration in glycosylation of mucins within the caecal niche of the parasite. Moreover, the mucins within the mucus barrier were less charged during chronic infection when compared to mice with acute infection. The presence of more highly charged mucins with an altered glycosylation pattern in acute infection may affect the physicochemical properties of the mucus barrier and contribute to worm rejection. For instance, mucin glycosylation may play an important role in the hydration of the mucus gel (30) and hence barrier properties of the mucus gel. Furthermore, mucin glycans are thought to provide a framework within the mucus gel, which allows the sequestration and presentation of essential host defence molecules (40).

In conclusion, in this study, we have characterised the changes within the dynamic mucosal barrier during acute and chronic nematode infection. The glycocalyx that coats the apical surface of the intestinal lumen was thickened in response to infection, with an increase in the cell surface mucins, *Muc4*, *Muc13* and *Muc17*. Moreover, we have clearly demonstrated that along with goblet cell hyperplasia observed during worm expulsion, there was an increase in the secretion of more highly charged mucins into the mucus barrier. Furthermore, during chronicity, the decrease in goblet cell numbers results in a depleted mucus barrier, containing predominantly lowly charged mucins. Taken together, this study highlights dynamic changes within the glycoconjugate components of the extrinsic and intrinsic mucosal barrier and emphasises that the quality and the quantity of these components may be important during worm expulsion.

## SUPPORTING INFORMATION

**Figure S1.** (a) BALB/c mice were infected orally with a high (acute infection) or a low dose (chronic infection) of *T. muris* eggs, and worm burdens were assessed on day 12, 14, 21, 35 and 42 pi. Levels of *IL-13* (b), *IL-4* (c) and *IFN*-γ (d) in the caecal tissue were determined using RT-PCR [data presented as fold change compared to naïve (red dashed line)]. Gross morphology (e) of the caecum of uninfected mice compared to during acute and chronic infection (day 35 pi.). H&E staining (f) of caecal tissue from mice with acute and chronic infection (day 35 pi.). The number of epithelial cells per crypt in 20 crypts was quantified (g). Quantification of the number of goblet cells (h) during the course of infection.

**Figure S2.** IL-4KO and IL-4R KO mice were infected with a high dose of *T. muris* eggs, and worm burdens (a) were assessed on day 35 pi. PAS staining (b) of the caecum from IL-4KO and IL-4R KO naïve mice and on day 18 and 32 pi.

**Figure S3.** Dual immunofluorescent staining with lectins (green) and mMuc2 antibody (red) in caecal tissue from mice with acute and chronic infection on days 21 and 35 pi.

**Table S1.** Forward and reverse primer sequences for the genes of interest.

**Table S2.** Levels of cell surface mucins in response to *T. muris* acute and chronic infection on day 14 and 35 after infection.
