# Supplementary figures and images for: Changes in the mucosal barrier during acute and chronic Trichuris muris infection
Source: Parasite Immunol. 2011 Jan;33(1):45–55. doi: 10.1111/j.1365-3024.2010.01258.x (PMC3020324; doi:10.1111/j.1365-3024.2010.01258.x)

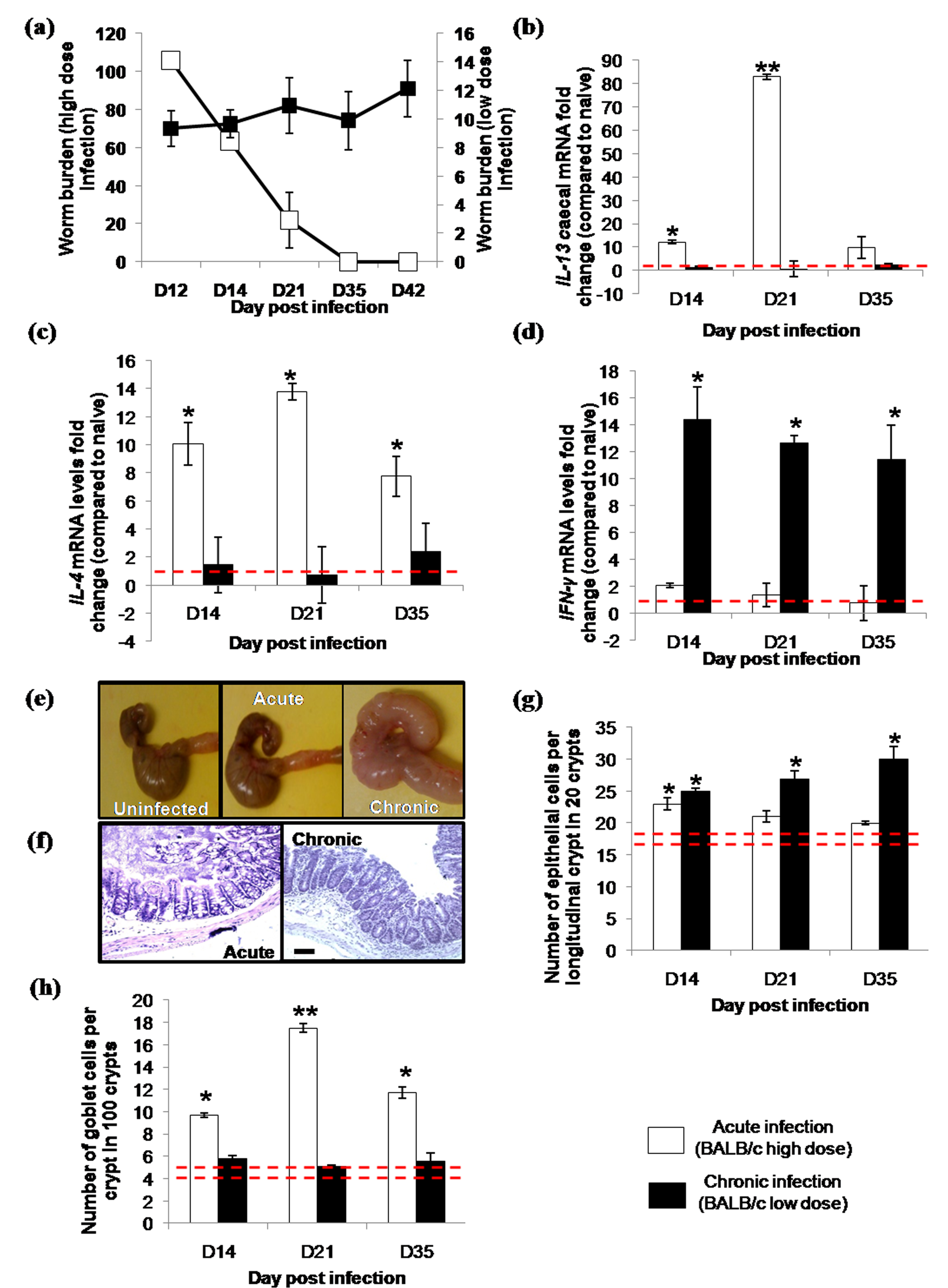

Supplement: Supplementary file 1 [file pim0033-0045-SD1.tif]

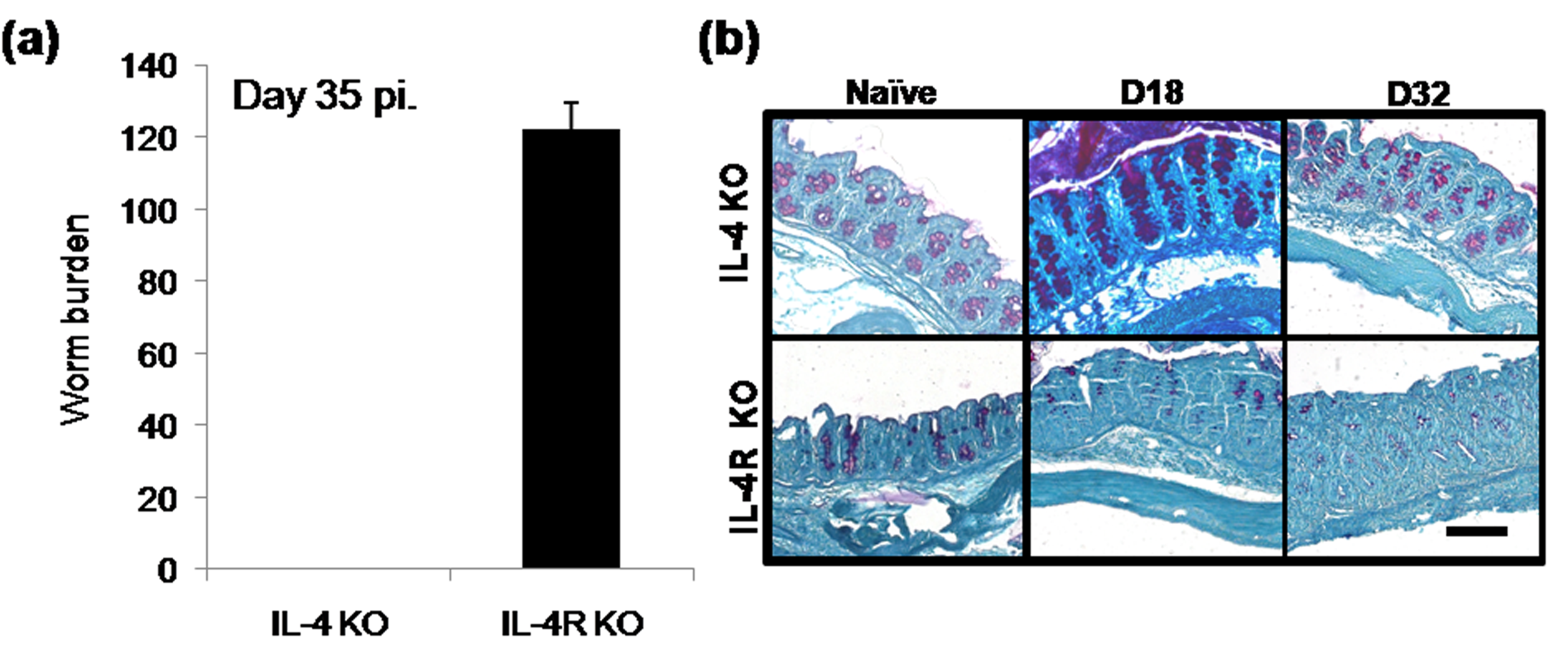

Supplement: Supplementary file 2 [file pim0033-0045-SD2.tif]

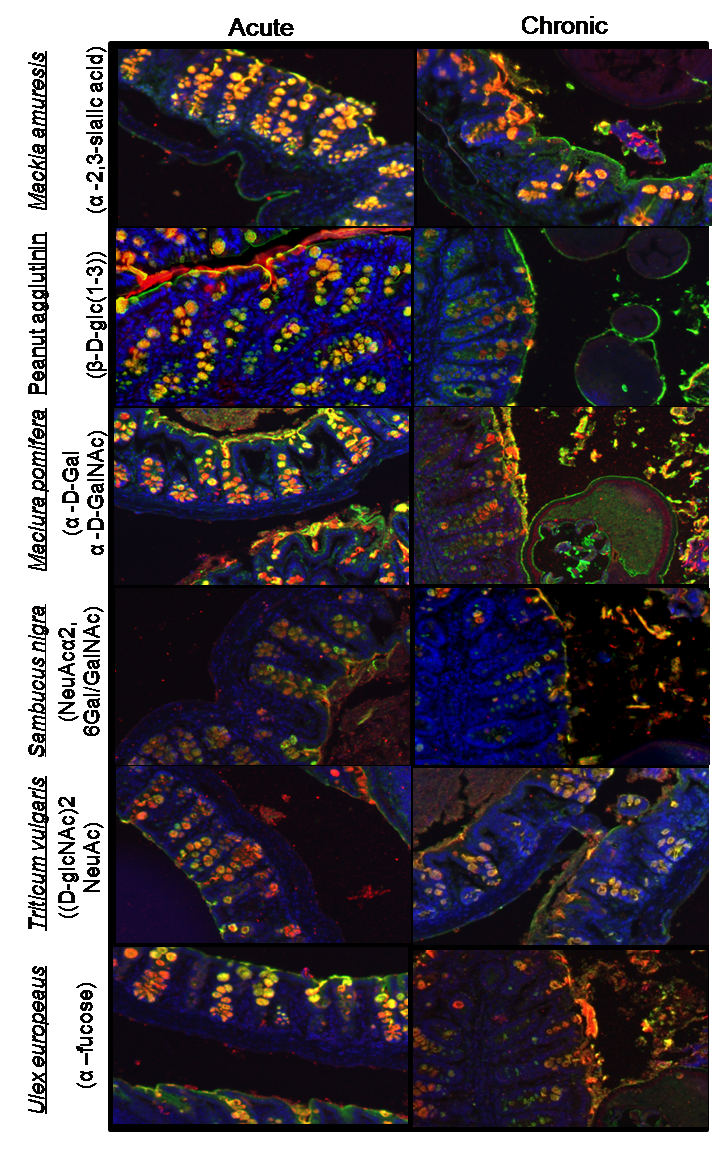

Supplement: Supplementary file 3 [file pim0033-0045-SD3.tif]

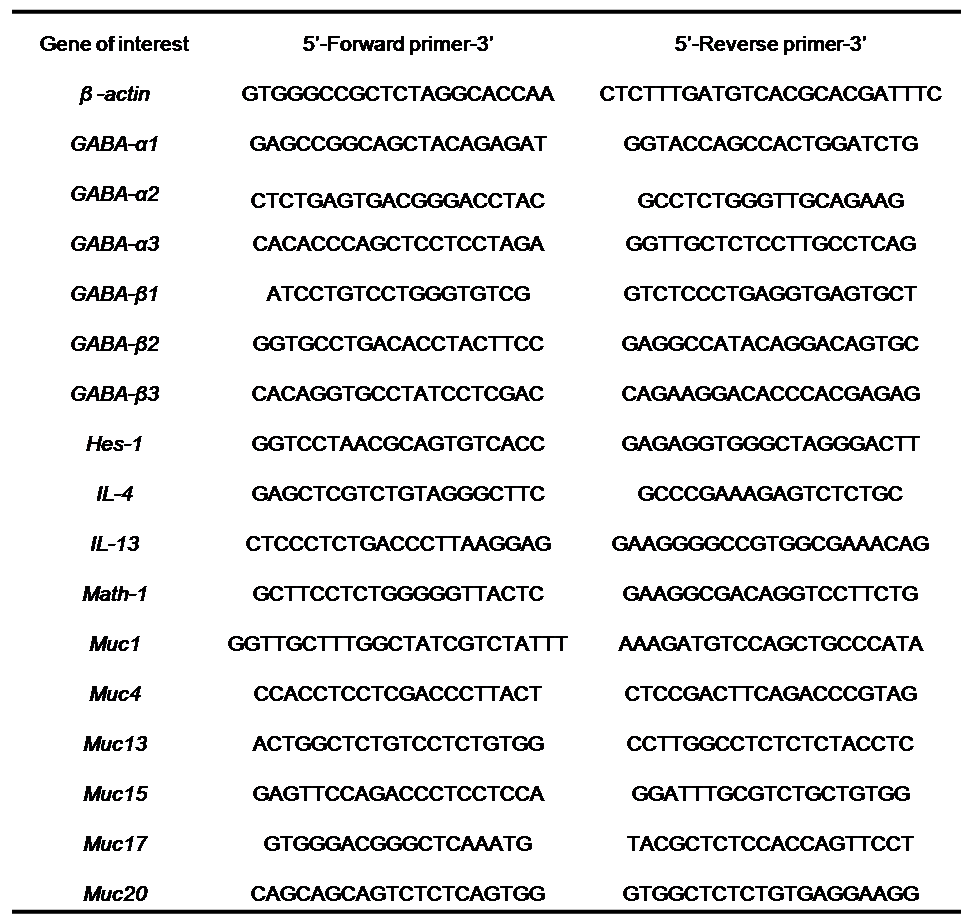

Supplement: Supplementary file 4 [file pim0033-0045-SD4.tif]

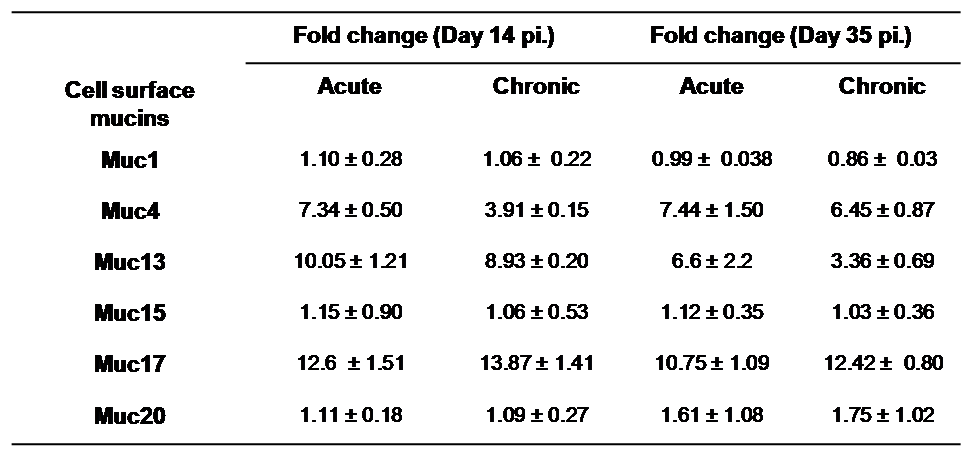

Supplement: Supplementary file 5 [file pim0033-0045-SD5.tif]
